# A deep learning-based computer-aided diagnosis method of X-ray images for bone age assessment

**DOI:** 10.1007/s40747-021-00376-z

**Published:** 2021-04-20

**Authors:** Shaowei Li, Bowen Liu, Shulian Li, Xinyu Zhu, Yang Yan, Dongxu Zhang

**Affiliations:** 1 Department of Children’s Health Care, Women and Children Hospital of Huli District, Xiamen, 361000 China; 2 State Key Laboratory of Molecular Vaccinology and Molecular Diagnostics, National Institute of Diagnostics and Vaccine Development in Infectious Diseases, School of Public Health, Xiamen University, Xiamen, 361102 China

**Keywords:** Bone age assessment, Computer-aided diagnosis, Unsupervised learning of object localization, Pre-trained image model

## Abstract

Bone age assessment using hand-wrist X-ray images is fundamental when diagnosing growth disorders of a child or providing a more patient-specific treatment. However, as clinical procedures are a subjective assessment, the accuracy depends highly on the doctor’s experience. Motivated by this, a deep learning-based computer-aided diagnosis method was proposed for performing bone age assessment. Inspired by clinical approaches and aimed to reduce expensive manual annotations, informative regions localization based on a complete unsupervised learning method was firstly performed and an image-processing pipeline was proposed. Subsequently, an image model with pre-trained weights as a backbone was utilized to enhance the reliability of prediction. The prediction head was implemented by a Multiple Layer Perceptron with one hidden layer. In compliance with clinical studies, gender information was an additional input to the prediction head by embedded into the feature vector calculated from the backbone model. After the experimental comparison study, the best results showed a mean absolute error of 6.2 months on the public RSNA dataset and 5.1 months on the additional dataset using MobileNetV3 as the backbone.

## Introduction

Skeletal development is a continuous variation process. It has distinct maturity markers that can be identified and analyzed by radiologists and pediatricians. Bone age is a quantitative metric of skeletal maturity [1]. The discrepancy between bone age and chronological age is closely related to physical development, such as body size, the appearance of the pubertal growth spurt, changes of sex characters, the level of endocrine hormone [2–7]. In clinical practice, this assessment is conducted by the pattern analysis of specific skeletal maturity markers on hand-wrist X-ray images.

**Fig.1:**
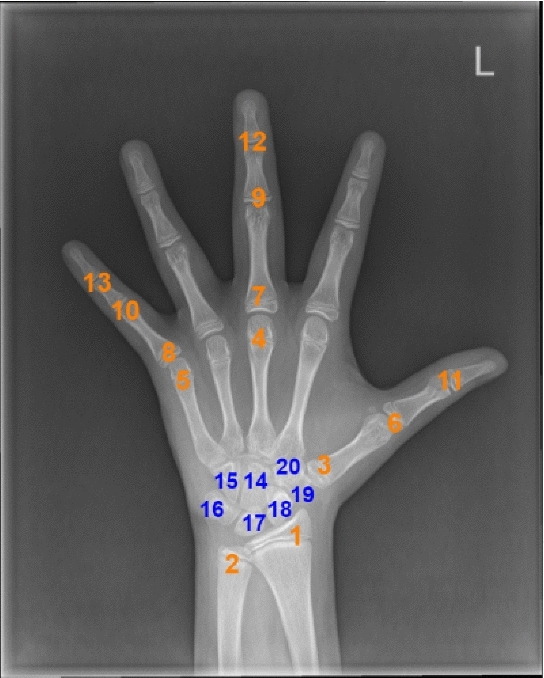
TW3 method divides the hand-wrist bones into two series: (1) radius, ulna, and part of short finger bones (RUS) and (2) carpal bones(Carpal). RUS: 1–13; Carpal: 14–20

Common clinical methods comprise the atlas method and the scoring method. A representative of atlas methods is the Greulich and Pyle (G&P) method [8], radiologists compared the X-ray image to be tested with atlas as a reference and used the closest match as the assessment result, which is simply and conveniently accomplished. However, the description of the stages of the bones should be more detailed and it is difficult to accurately determine bone age when the X-ray image to be tested is between two adjacent reference atlas. In Tanner–Whitehouse (TW) method, which is a scoring method, radiologists first focused on 20 specific regions of interest (ROIs) and then analyzed them individually to evaluate bone age [9]. Through revisions and updates, the latest version of the TW method is called TW3 for short. 20 ROIs considered in TW method are shown in Fig. 1. More indicators and parameters are used in the TW3 method to achieve a more detailed description of skeletal development and improve the accuracy of bone age assessment. However, clinical procedures are a subjective and time-consuming assessment. As a result, evaluation results are hard to maintain an acceptable error margin in the modern radiology department.

Motivated by this and to meet the less-time tendency, a deep learning-based computer-aided diagnosis method was proposed for bone age assessment. Experimental investigation in this study shows that deep learning is sufficiently potential to create a fully automated bone age assessment system with accurate and authentic results.

## Related works

Some existing computer-aided methods were examined to find out which factor facilitated or hindered the availability of clinical practice. Most computer-aided bone age assessment methods can be divided into two categories: non-deep-learning methods and deep-learning methods. The former mainly used image processing technology and classic machine learning [10]. For example, Pietka et al. [11] used different window sizes with adaptive thresholds to discriminate bone tissue and other regions. In terms of geometrical description and properties of pixel values, ROI was used to generate feature descriptors. Finally, the obtained feature descriptors were fed into decision-making methods to output an estimation of bone age. The extraction of the epiphyseal and metaphyseal tissues was illustrated in [12]. Feature descriptors were obtained by the diameter of the critical bone area and the ratios of the crucial distance measurement. BoneXpert [13], as a commercial automated method, implemented a generative model to generated images with retaining realistic shapes and densities, collectively denoting bone appearance. Features included shape, intensities, and texture information. This method implemented an automated assessment by mapping functions to give a relative score depends on choosing the TW or G&P methods. However, images with poor quality or an abnormal bone structure will be rejected, thus the process could be manual sometimes. Non-deep-learning methods commonly utilized hand-crafted visual features from the whole images or local informative regions and the classifiers were developed on a private and small-scale dataset. The results ranged from 10 to 28 months mean absolute error (MAE) and were easily affected by hand-wrist X-ray images with unexpected image quality. The generalization ability of those models is questionable.

In deep-learning methods, BoNet [14] utilized a purpose-designed Convolutional Neural Network (CNN) to extract low and middle-level feature descriptors and employed an additional deformation layer to account for nonrigid object deformation. Finally, fully connected layers were implemented to accomplish bone age assessment. BoNet achieved a MAE of about 9.6 months. To promote the creation and development of machine learning models in analyzing medical images, a large-scale bone age assessment dataset, which consists of 12,611 images with various resolutions, was introduced by the Radiological Society of North America (RSNA) [15]. Data-processing, comprised of many subtasks, was undoubtedly a necessary procedure to located informative regions. In [16], both deep learning and classic machine learning were used to produce a reliable prediction. For the deep-learning-based method, pre-trained CNNs were implemented to extract image features automatically and build a regressor model. For the classic machine learning method, canny edge detection was implemented for feature extraction and there were five traditional machine learning regressors: Linear Regression, Random Forest, Support Vector Regressor (SVR), XGBoost, and Multilayer Perceptron (MLP). Finally, the mean absolute error achieved by pre-trained CNNs was the best result, 14.78 months. In [17], the author firstly trained the U-Net model to obtain key point regions with manually labeled hand masks. Subsequently, a key point detection model was applied to align hand radiographs into a common coordinate space. As a result, they achieved a 6.30 months MAE for males and a 6.49 months MAE for females. [18] proposed a novel experimental architecture with manually labeled bounding boxes and key point annotations during training. To perform pose estimation and region detection, local information was exploited for bone age assessment. As a result, they achieved the best in RSNA, 4.14 months MAE. Although the specific applications of many existing high-performing models showed high accuracy and efficiency with precisely manual annotations, extra annotations were time-consuming and hindered the transformation of algorithms to clinical applications.

In conclusion, the current methods show that deep-learning-based methods are being actively applied and can automatically generate feature descriptors representing the pattern of hand-wrist X-ray images. CNN has been the paradigm of choice in a wide range of computer vision image applications, resulting in a growing interest in application-specific features instead of hand-craft features. Most existing methods based on deep learning evaluated bone age by annotating extra bounding boxes or key points, even though manual annotations are expensive and subjective. In this study, hand region were first located using a complete unsupervised learning method and an image-processing pipeline was proposed. Subsequently, the image model with pre-trained weights was utilized as a backbone to enhance the reliability of prediction. The prediction head was implemented by a Multiple Layer Perceptron with one hidden layer. In compliance with clinical studies, gender information was an additional input to the prediction head by embedded into the feature vector calculated from the backbone model. The proposed method was developed on the RSNA dataset and the additional dataset, respectively. Lots of contrast experiments were implemented to demonstrate the superiority of the proposed method and the significance of gender-embedding.

The rest of this paper is structured as follows: “Dataset and image-processing” presents the description of the public RSNA dataset and the additional dataset. The unsupervised learning framework of object localization and purpose-designed image pre-processing pipeline are also illustrated. In “Deep-learning-based bone age assessment”, an image model with pre-trained weights and a Multiple Layer Perceptron were implemented to accomplish bone age assessment. The superiority of the proposed method and the significance of gender-embedding were demonstrated by lots of contrast experiments. “Conclusion” provides the analysis of elements that facilitated or hindered the availability of clinical use and discussion about future research.

## Dataset and image-processing

This section describes attributes of the datasets employed in this study, informative regions localization based on a complete unsupervised learning method, and a purpose-designed image-processing pipeline.

### Dataset

**Fig. 2:**
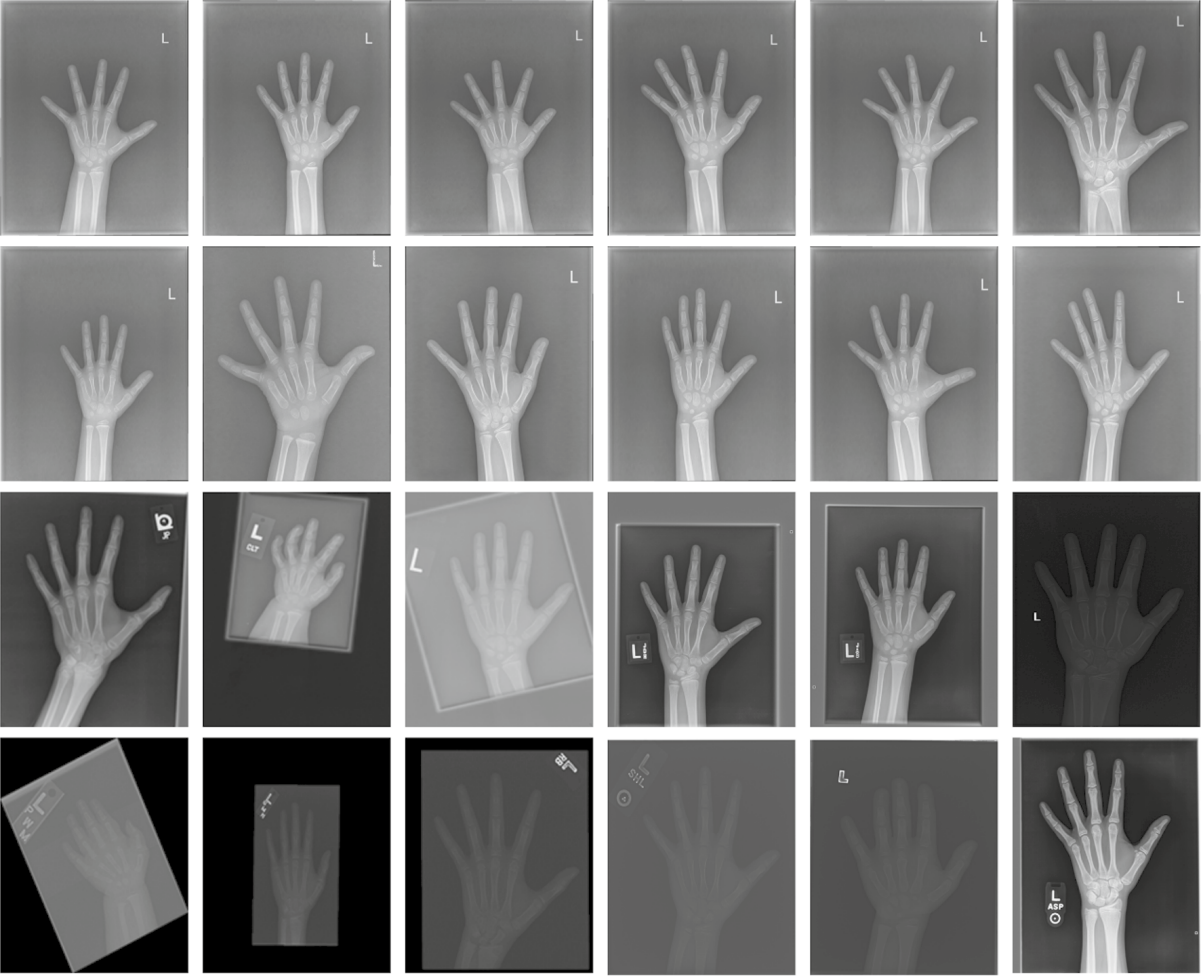
Samples from the dataset. Images in the first two rows are from the additional dataset. The remaining part is from RSNA dataset

The proposed method was developed on the public RSNA dataset of 12,611 X-ray images and an additional dataset of 1709 images separately. The additional dataset is from the Department of Children's Health Care, Women and Children Hospital of Huli District, Xiamen. As shown in Fig. 2, there are samples from the dataset. The attributes of the dataset are as follows:

1. ID: the ID of the image.
2. bone age: the bone age of each image, i.e., the ground truth.
3. male: the gender information (male: True, female: False).

**Fig. 3:**
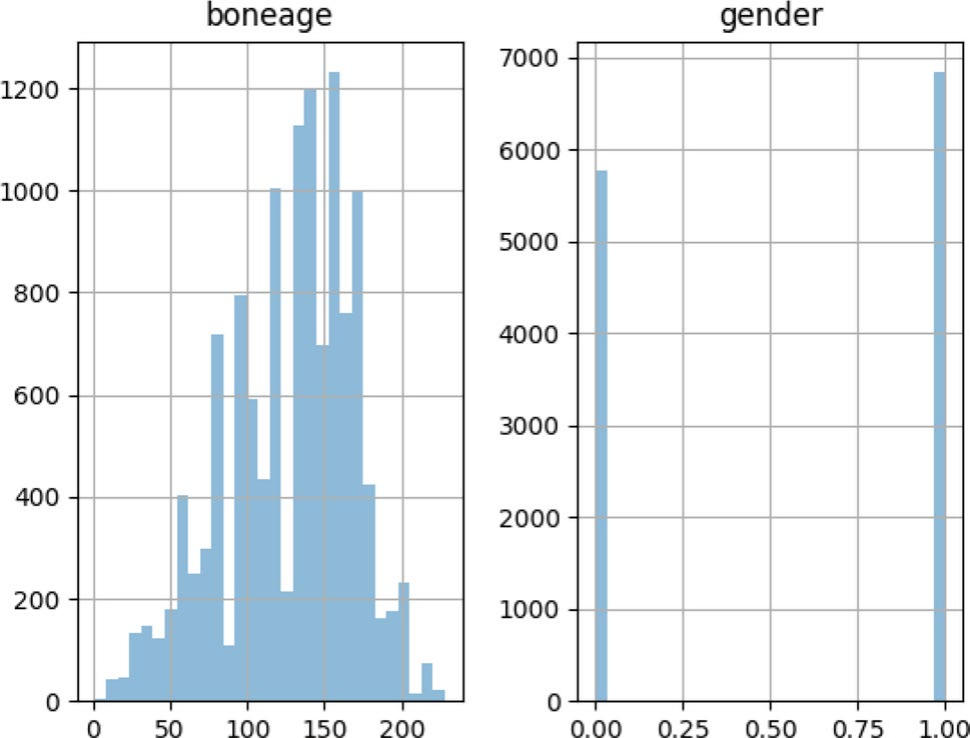
The distribution of bone age and gender in the RSNA dataset

**Fig. 4:**
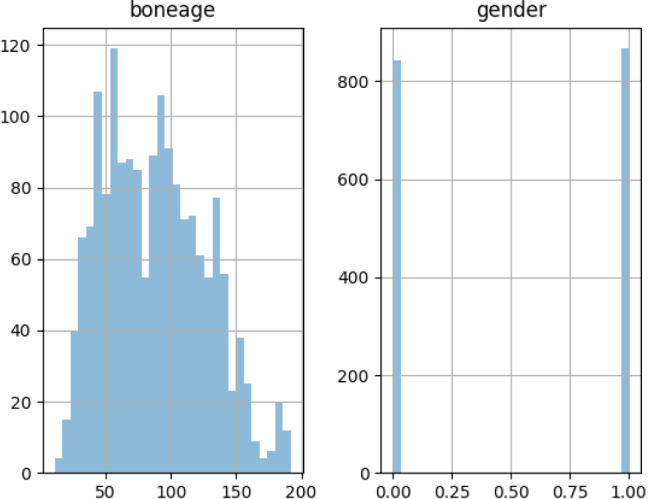
The distribution of bone age and gender in the additional dataset

The distribution of bone age and gender in the RSNA dataset and the additional dataset is shown in Figs. 3 and 4. Based on a 7:2:1 training-validation-testing ratio, two datasets were split individually.

### Unsupervised learning of object localization

**Fig. 5:**
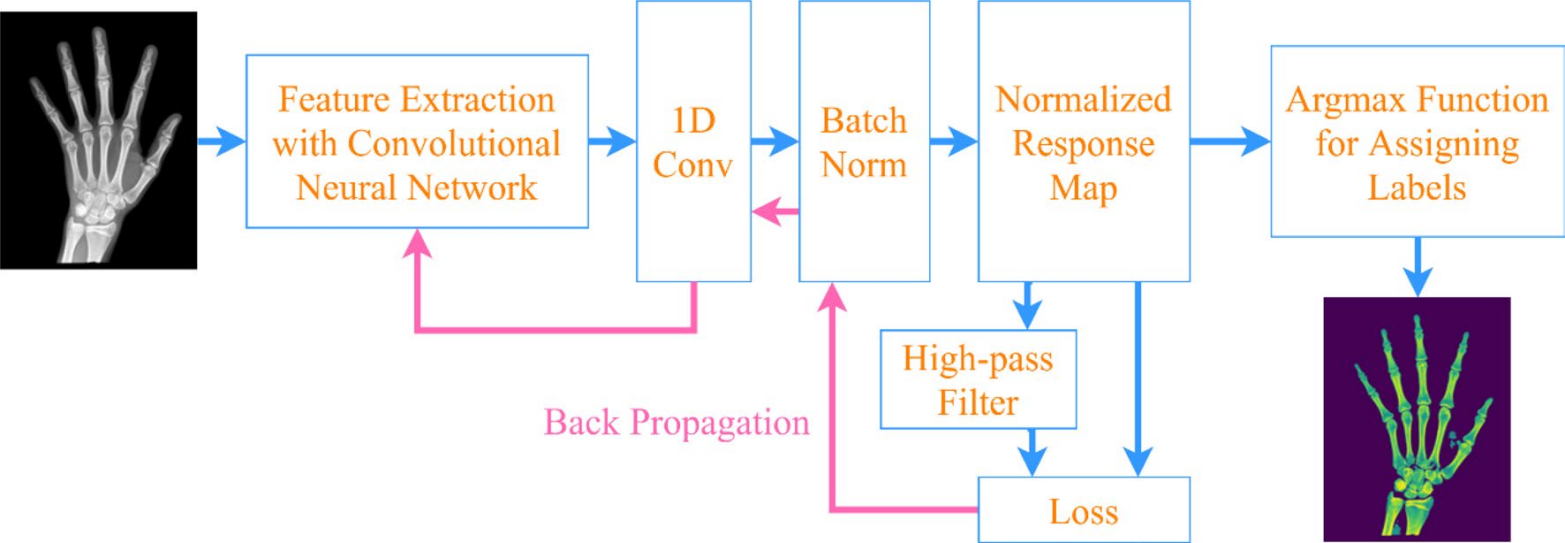
Illustration of the unsupervised method

Due to the various resolutions of X-ray images in the dataset, it is necessary to locate object regions to enhance informative areas and suppress noise. Therefore, an unsupervised learning framework, which was inspired by a novel unsupervised learning image-segmentation approach proposed in [19], was exploited to accomplish it. Supervised methods require the original image and ground truth with pixel-level semantic labels. However, in unsupervised methods, there is no training image or ground truth with labels of pixels. It assigns labels to pixels assuming that an image is divided into differentiable regions without any previous knowledge. The framework can implement object detection based on differentiable feature clustering. CNN was utilized to obtain pixel-level feature descriptors from the content of the target image. Those pixel-level feature descriptors could be sufficiently discriminative to recognize desired object region. The mechanism of this framework, as shown in Fig. 5, consists of convolutional layers to extract high-level features, a batch normalization layer of those high-level features, and an argmax layer for differentiable feature clustering. Finally, the backpropagation of the proposed loss function accomplished image segmentation in an unsupervised way.

A detailed illustration is as follows. In an input image $X \in {\mathbb{R}}^{C \times H \times W}$, $\left\{ {x_{n} \in {\mathbb{R}}^{C} } \right\}$ denotes one of the pixels in the input image. For $\left\{ {x_{n} \in {\mathbb{R}}^{C} } \right\}$, $p$ dimensional feature descriptor $\left\{ {r_{n} \in {\mathbb{R}}^{p} } \right\}$ was first obtained from the convolutional layers and $p$ denotes the number of channels of Convolutional filters. Subsequently, the feature descriptor is fed into a one-dimensional (1D) convolutional layer, where the number of channels produced by the convolution is q and the size of the convolution kernel is $1 \times 1$, then is normalized using batch normalization. As a result, the response $\left\{ {y_{n} \in {\mathbb{R}}^{q} } \right\}$ was obtained and $q$ denotes the upper bound of the possible amounts of unique labels. Finally, an argmax function was utilized to accomplish label assigning: The cluster label for the pixel $x_{n}$ was denoted as $c_{n}$, which was equal to the dimensional of the maximum value of $y_{n}$.

For the whole structure, two-loss functions, which described below, are utilized as a strategy to make $q$ descending and obtain the optimal parameters of the network. The details of the two kinds of loss functions are described as follows. The loss function $L$, which consists of similarity loss and spatial continuity loss, is denoted as follows:

1 $$ L = \lambda L_{sim} \left( {\left\{ {y_{n} ,c_{n} } \right\}} \right) + \mu L_{con} \left( {\left\{ {y_{n} } \right\}} \right), $$

where $\lambda$ and $\mu$ represent the weights to balance these two constraints.

The constraint on the feature similarity is

2 $$ L_{{{\text{sim}}}} (\{ y_{n} ,c_{n} \} ) = \sum\limits_{n = 1}^{N} {\sum\limits_{i = 1}^{q} { - \delta (i - c_{n} )} } ln(y_{n,i} ), $$

and

3 $$ \begin{gathered} N = H \times W, \hfill \\ \delta (t) = \left\{ \begin{gathered} 1,{\text{ if }}t = 0 \hfill \\ 0,{\text{ otherwise}} \hfill \\ \end{gathered} \right. \hfill \\ \end{gathered} $$

$\left\{ {y_{n,i} } \right\}$ denotes the $i \, th$ element of $\left\{ {y_{n} } \right\}$. When considering feature similarity, the elements in the response vector will be assigned to corresponding labels according to the above principle.

The regulation on spatial continuity is

4 $$ L_{{{\text{con}}}} (\{ y_{n} \} ) = \sum\limits_{\xi = 1}^{W - 1} {\sum\limits_{\eta = 1}^{H - 1} {\left\| {y_{\xi + 1,\eta } - y_{\xi ,\eta } } \right\|_{1} } } + \left\| {y_{\xi ,\eta + 1} - y_{\xi ,\eta } } \right\|_{1} $$

where the L1-norm was used as criterion and differences of the response vector in horizontal and vertical directions was considered as a distance regulation. Here, $W$ and $H$ denotes the width and height of an input image, $y_{\xi ,\eta }$ denotes the pixel value at $(\xi ,\eta )$ in the response vector $y_{n}$.

Aimed to overcome the impact of various resolution, each target image was fed into the network individually, therefore, parameters will update and optimize based on the content of the current image. As mentioned above, for a target image, the forward process of the network predicts the cluster labels and the backward process, that is, calculating and backpropagating the loss $L$. It updates the parameters using a stochastic gradient descent with a momentum of 0.9 and learning rate set to 0.1. Two convolutional layers were used to obtain a feature descriptor and $p = q = 90$ for initialization. The number of iterations was set to 50. Notably, to balance loss functions exploited in this study, different values of $\lambda$ and $\mu$ were set manually during the experiments. The results show that $\lambda = 4$ and $\mu = 1$ are the best when applied to image segmentation.

### Image processing pipeline

**Fig. 6:**
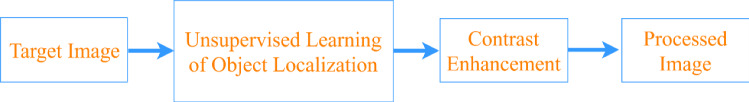
Proposed image-processing pipeline

**Fig. 7:**
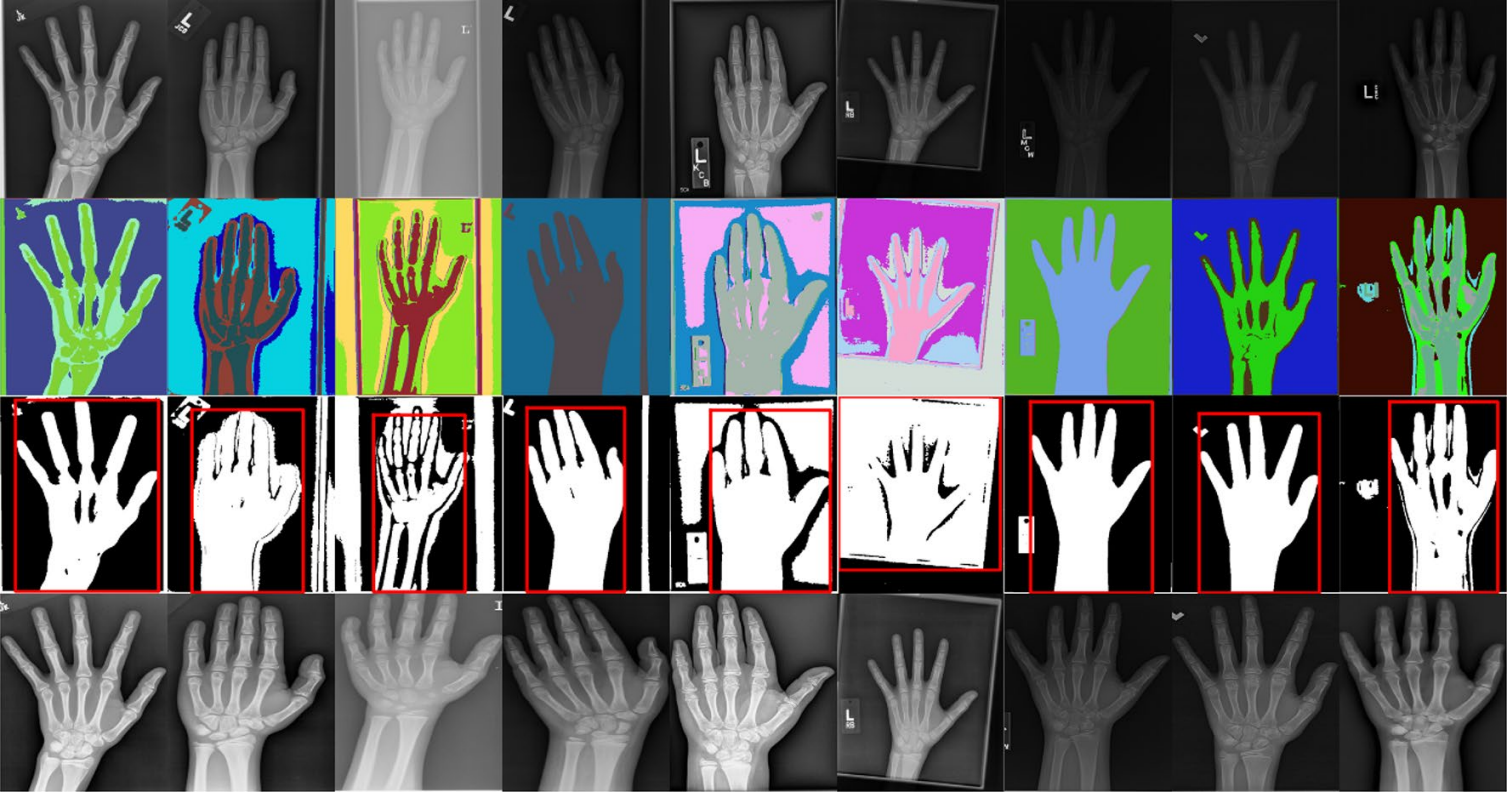
Original images (first row), segmentation results (second row), generating bounding box of the hand region (third row), and the final result (last row)


The abovementioned method distinguished informative object regions and other regions, which is difficult for general threshold segmentation algorithms. However, due to various resolution and contrast in the dataset, segmentation results cannot provide masks with a clean and sharp edge. The background was first removed and the region with the maximum area was regarded as the hand region. Then, the target image was cropped based on the bounding box of the informative region through connectivity analysis on the segmentation result. The pseudocode for the unsupervised object localization algorithm is shown in Algorithm 1. The proposed image pre-processing pipeline is shown in Fig. 6 and the results of each step are shown in Fig. 7.

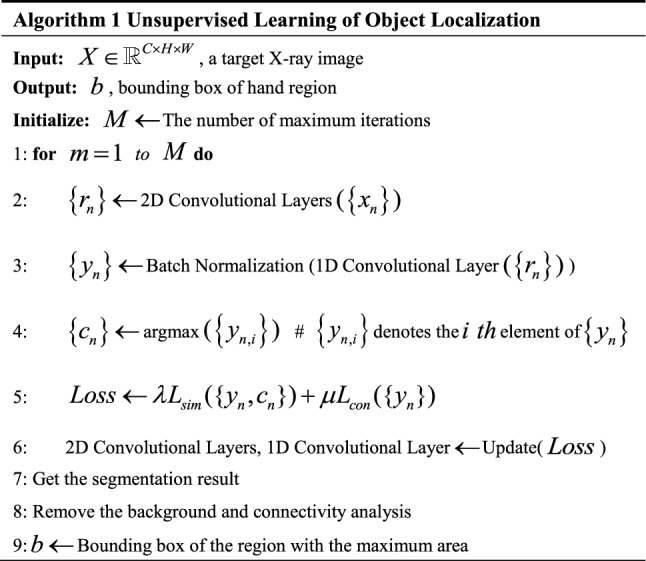


## Deep-learning-based bone age assessment

CNNs have been the paradigm of choice in modern artificial intelligence, resulting in a growing interest in application-specific features instead of hand-craft features. Multiple Layer Perceptron (MLP) can distinguish data that are not linearly separable and map feature descriptors calculated from CNNs onto output with desired dimensions. Most deep learning-based methods evaluated bone age by extra bounding boxes or key points, despite expensive and subjective annotations. Based on unsupervised learning of object localization and the proposed image-processing pipeline, images with a large area of informative regions were obtained. In this study, to enhance the reliability of prediction, an image model with pre-trained weights was used as the backbone and the prediction head was implemented by a Multiple Layer Perceptron with one hidden layer. Besides, gender information was an additional input to the prediction head by embedded into the feature vector calculated from the backbone model. Lots of contrast experiments was performed to demonstrate the superiority of the proposed method and the necessity of gender-embedding.

### Outline of MobileNetV3

**Fig. 8:**
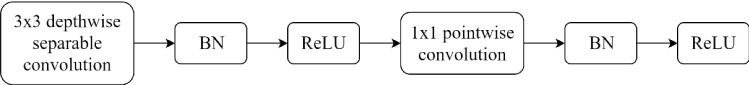
The structure of Depthwise Separable Convolution

MobileNetV3 [20] was built based on more efficient building blocks to improve the speed-accuracy tradeoff. MobileNetV3 was proposed on the basis of MobileNetV1 [21] and MobileNetV2 [22]. The main modules of MobileNetV3 are as follows:

A. Depthwise Separable Convolution: In [21], depthwise separable convolutions can reduce the model size and build lightweight deep neural networks. Depthwise separable convolutions, as shown in Fig. 8, are comprised of two layers: a depthwise convolution, as spatial filtering, to apply an individual filter to each channel of input and a 1 × 1 pointwise convolution to combine features from the depthwise convolution and change the number of channels in the feature map.

**Fig. 9:**
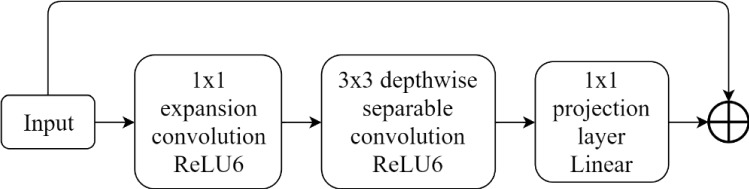
The structure of inverted residual block

B. Inverted Residual Block: MobileNetV2 introduced an inverted residual block as an even more efficient linear bottleneck with a residual structure for reference. The inverted residual block, as shown in Fig. 9, is comprised of a 1 × 1 expansion convolution to obtain better results without an excessive calculation, depthwise convolutions, and a 1 × 1 projection layer. Because of a residual connection, the input and output are connected when they have the same channels. This structure generates a compact feature descriptor as the output, and at the same time expands to a feature space with a higher dimension to improve the ability to obtain nonlinear relationships.

**Fig. 10:**
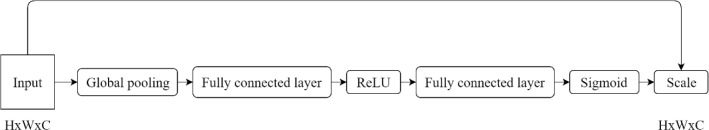
The structure of SE block

C. Squeeze-and-Excitation [23]: As shown in Fig. 10, this module is used to model the dependence between the feature channels, and automatically obtain the importance of each feature channel through training. As a result, it can improve the useful features and suppress the uselessness of the current task. The first is the Squeeze operation. Feature compression is performed along the spatial dimension. Each two-dimensional feature channel is turned into a scalar which has a global receptive field to some extent and the output dimension equals the number of input feature channels. It characterizes the global distribution of the response on the feature channels and enables the layer close to the input to obtain the global receptive field. Secondly, the Excitation operation generates weights for each feature channel, where weights are updated to explicitly model the correlation between the feature channels. Finally, the Reweight operation weights feature channels through multiplication to accomplish recalibration of original features. In detail, global average pooling is used as the Squeeze operation to reduce feature dimensions. Then two Fully Connected layers form a Bottleneck structure is used to model the correlation between channels and output the same number of weights as the input channels. Between these two layers, the activation function is implemented by ReLU. The advantages of using two Fully Connected layers are: (1) It has more nonlinearity and can better fit the complex correlation between channels; (2) It greatly reduces the number of parameters and calculations. Then a Sigmoid function outputs the normalized weights between 0 and 1, and finally a Scale operation weights the normalized weight to the features of each channel.

**Fig. 11:**
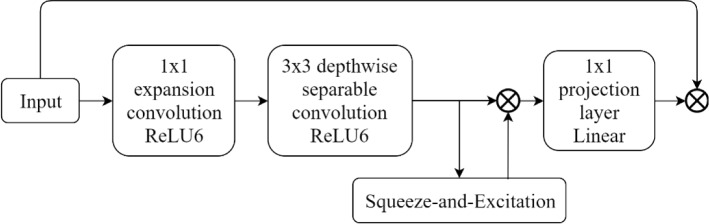
The structure of MobileNetV3 block

D. As shown in Fig. 11, the MobileNetV3 block was introduced based on the analysis of the abovementioned structures. To significantly improve the accuracy of neural networks, the h-swish (the hard version of swish) was proposed to replace the Sigmoid function.

5 $$ h - {\text{swish}}(x) = x\frac{{{\text{ReLU}} (x + 3)}}{6}. $$

### Details of training

**Fig. 12:**
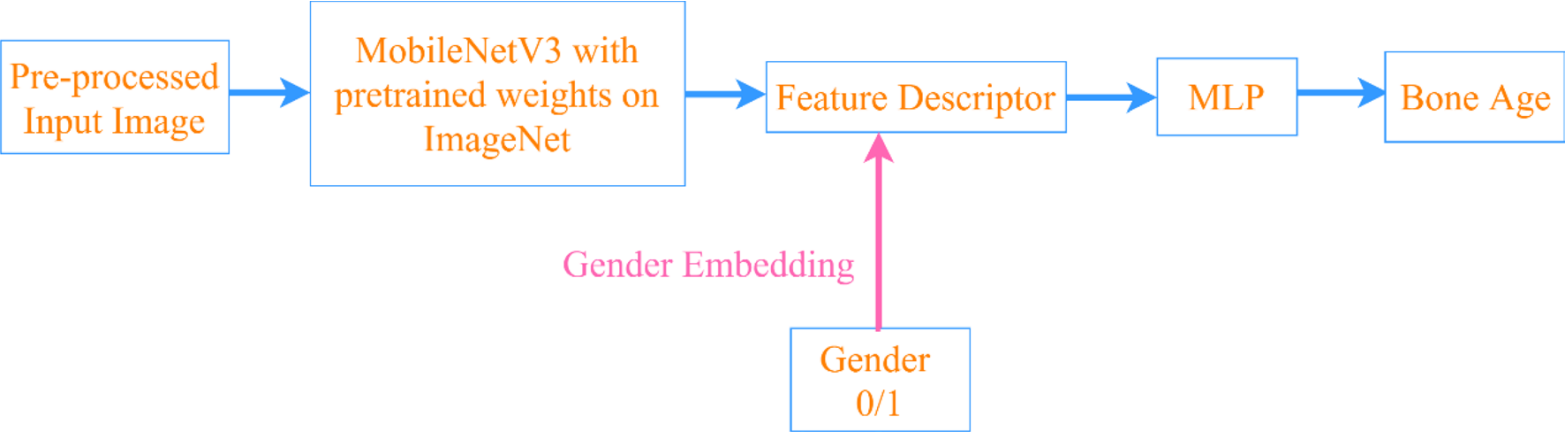
The workflow of the proposed model

The network was trained on the RSNA dataset and the additional dataset, respectively. As shown in Fig. 12, MobileNetV3 with pre-trained weights on ImageNet was utilized as the backbone and the prediction head was implemented by a Multiple Layer Perceptron with one hidden layer. A linear transformation was applied to the gender information (0/1) and the size of the output was set to 24. After a ReLU function, it was regarded as an additional input to the prediction head by embedded into the feature vector calculated from the backbone model. The hidden layer in the Multiple Layer Perceptron consisted of 1304 neurons and ReLU was used as the activation. The overall architecture of the MobileNetV3 as the backbone and the prediction head used in this study was shown in Table 1.

**Table 1:** Overall architecture of the MobileNetV3 as backbone and the prediction head

Input	Operator	Out	SE	NL	Stride
224 × 224 × 3	Conv 2d, 3 × 3	16	False	HS	2
112 × 112 × 16	3 × 3	16	False	RE	1
112 × 112 × 16	3 × 3	24	False	RE	2
56 × 56 × 24	3 × 3	24	False	RE	1
56 × 56 × 24	5 × 5	40	True	RE	2
28 × 28 × 40	5 × 5	40	True	RE	1
28 × 28 × 40	3 × 3	40	True	RE	1
28 × 28 × 40	3 × 3	80	False	HS	2
14 × 14 × 80	3 × 3	80	False	HS	1
14 × 14 × 80	3 × 3	80	False	HS	1
14 × 14 × 80	3 × 3	80	False	HS	1
14 × 14 × 80	3 × 3	112	True	HS	1
14 × 14 × 112	3 × 3	112	True	HS	1
14 × 14 × 112	5 × 5	160	True	HS	2
7 × 7 × 160	5 × 5	160	True	HS	1
7 × 7 × 160	5 × 5	160	True	HS	1
7 × 7 × 160	Conv2d, 1 × 1	960	False	HS	1
7 × 7 × 960	AvgPool	960	False	–	1
1 × 1 × 960	Conv2d, 1 × 1, NBN	1280	False	HS	1
1 × 1 × 1280	Gender Embedding	1304	False	RE	–
1 × 1304	MLP	Prediction	–	–	–

“Input” stands for the size of the input, Height × Weight × Channel. In “Operator” column, “Conv2d, 3 × 3” indicates traditional convolution layer and the size of the convolution kernel, “3 × 3” means 3 × 3 depthwise separable convolution in MobileNetV3 block, “NBN” denotes no batch normalization and “AvgPool” stands for applying a 2D adaptive average pooling over an input signal composed of several input planes. “Out” means the number of channels in the output. “SE” denotes whether there was a Squeeze-And-Excite in that block. “NL” denotes the type of nonlinearity used. Here, “HS” denotes h-swish and “RE” denotes ReLU. “Stride” means the stride for the cross-correlation in a 2D Convolution

During training, data augmentation, such as cropping, resizing, rotation, and transformation in contrast and brightness, was applied to the input X-ray images to prevent the problem of overfitting. Overall architecture of the MobileNetV3 as the backbone and the prediction head is described in Table 1 and the number of parameters of the architecture is approximately 7.6 Million. The learning rate was set to the initial learning rate decayed by 10 times every 30 epochs. The Batch size was set to 10 and the whole network was trained for 160 epochs. The stochastic gradient descent optimizer with a momentum of 0.9 was used to minimizing the mean squared error (squared L2 norm) between each element in the prediction and the target. The loss function is:

6 $$ {\text{MeanSquaredError}} = \frac{1}{N}\sum\limits_{i = 1}^{N} {(\hat{Y}_{i} - Y^{\prime}_{i} )^{2} } , $$

where $N$ is the number of the training set, $\hat{Y}_{i}$ is the output of the model and $Y_{i}^{\prime }$ is the bone age after standardization:

7 $$ Y_{i}^{\prime } = \frac{{Y_{i} - \mu }}{\sigma }, $$

where $Y_{i}$ is the original value of the bone age (month), $\mu$ is the mean of the bone age and $\sigma$ is the standard deviation of the bone age.

### Performance of the proposal

The mean absolute error was applied to evaluate the performance of the proposed method. As shown in Table 2, lots of contrast experiments were implemented to evaluate the performance in different conditions. Obviously, gender-embedding and image-processing are helpful for the performance of the network. The model with image size set to 700 $\times$ 700, gender-embedding and image-processing achieved the best result. Besides, as shown in Table 3, the method was compared with other methods mentioned in “Related works” and those methods were all developed on the public RSNA dataset. Although many existing high-performing models showed high accuracy and efficiency with precisely manual annotations, extra annotations were time-consuming and hindered the transformation of algorithms to clinical applications. Without training annotations, the method achieves acceptable results without requiring manual annotation.

**Table 2:** Experimental results in different conditions

Dataset	Gender	Pre-process	Pre-trained	Image Size	MAE(months)
RSNA	True	True	True	224 × 224 × 3	6.8
	True	True	True	500 × 500 × 3	6.5
	True	True	True	700 × 700 × 3	**6.2**
	False	True	True	700 × 700 × 3	8.5
	True	False	True	700 × 700 × 3	7.5
	True	True	False	700 × 700 × 3	7.4
Additional Dataset	True	True	True	224 × 224 × 3	5.7
	True	True	True	500 × 500 × 3	5.6
	True	True	True	700 × 700 × 3	**5.1**
	False	True	True	700 × 700 × 3	6.0
	True	False	True	700 × 700 × 3	6.2
	True	True	False	700 × 700 × 3	5.9

MobileNetV3 with pre-trained weights was exploited as the backbone. “Gender” indicates whether there was gender-embedding. “Pre-process” stands for the input X-ray images were original or processed by the framework described in “Dataset and image-processing”. “Pre-trained” means whether pre-trained backbone was used. “Image size” indicates the size of input X-ray images

**Table 3:** Comparison the method proposed in this study with other methods mentioned in “Related works” and following methods were developed on RSNA dataset

Method	Manual annotation	MAE (months)
[18]	Bounding box and keypoint	4.14
[17]	Mask and keypoint	6.4
[16]	No	14.78
Method in this study	No	6.2

## Conclusion

In this study, a deep learning-based computer-aided diagnosis method was proposed for performing bone age assessment. To reduce expensive manual annotations, informative region was firstly located based on a complete unsupervised learning method and an image-processing pipeline was proposed. The unsupervised learning method consists of convolutional layers to extract high-level features, a batch normalization layer of those high-level features and an argmax layer for differentiable feature clustering. The backpropagation of the proposed loss function accomplished image segmentation in an unsupervised way. As a result, it can distinguish informative object region and other regions without manual annotations. Subsequently, the background was removed and the region with the maximum area was regarded as the hand region and target images were cropped based on the bounding box of the informative region through connectivity analysis on segmentation results. Finally, histogram equalization was applied to adjust contrast. To produce a reliable prediction, image models with pre-trained weights was exploited as the backbone and the prediction head was implemented by a Multiple Layer Perceptron. In compliance with clinical studies, gender information was an additional input to the prediction head by embedded into the feature vector calculated from the backbone model. After experimental comparison of various model architectures, the best results showed a mean absolute error of approximately 6.2 months on the RSNA dataset and 5.1 months on the additional dataset using MobileNetV3 as the backbone.

To facilitate the availability of clinical use and provide more accurate results, fine-grained local information which is the most relevant to the prediction should be extracted without extra manual annotations. In a future study, a purpose-designed deep learning framework will be proposed to extract more fine-grained features and create an automated application with accuracy comparable to that of radiologists and pediatrician.

## Data Availability

The dataset employed in this paper is private, it is not available for now.

## Code availability

All code was implemented in Python. Both the code and scripts to reproduce the experiments of this paper will be available soon.

## References

[1] Dallora AL, Anderberg P, Kvist O, Mendes E, Diaz Ruiz S, Sanmartin Berglund J (2019). Bone age assessment with various machine learning techniques: a systematic literature review and meta-analysis. PLoS ONE.

[2] Marshall WA (1974). Interrelationships of skeletal maturation, sexual development and somatic growth in man. Ann Hum Biol.

[3] Hägg U, Taranger J (1980). Skeletal stages of the hand and wrist as indicators of the pubertal growth spurt. Acta Odontol Scand.

[4] Hauspie R, Bielicki T, Koniarek J (1991). Skeletal maturity at onset of the adolescent growth spurt and at peak velocity for growth in height: A threshold effect?. Ann Hum Biol.

[5] Marshall WA, Limongi Y (1976). Skeletal maturity and the prediction of age at menarche. Ann Hum Biol.

[6] Thompson GW, Popovich F, Luks E (1973). Sexual dimorphism in hand and wrist ossification. Growth.

[7] Lee H, Tajmir S, Lee J, Zissen M, Yeshiwas BA, Alkasab TK, Choy G, Do S (2017). Fully automated deep learning system for bone age assessment. J Digit Imaging.

[8] Greulich WW, Pyle SI (1959) “Radiographic atlas of skeletal development of the hand and wrist.” Am J Med Sci 238. 10.1097/00000441-195909000-00030

[9] Goldstein H, Cameron N, Healy J M, Tanner M (2001) Assessment of Skeletal Maturity and Predication of Adult height (TW3 Method). Government \& Opposition 36(1):27–47.

[10] Mansourvar M, Ismail MA, Herawan T, Raj RG, Kareem SA, Nasaruddin FH (2013) Automated bone age assessment: Motivation, taxonomies, and challenges. Comp Math Methods Med 2013:391626. http://www.ncbi.nlm.nih.gov/pubmed/391626-391626 10.1155/2013/391626 PMC3876824 24454534

[11] Pietka E, Kaabi L, Kuo ML, Huang HK (1993). Feature extraction in carpal-bone analysis. IEEE Trans Med Imaging.

[12] Pietka E, Gertych A, Pospiech S, Cao F, Huang HK, Gilsanz V (2001). Computer-assisted bone age assessment: Image preprocessing and epiphyseal/metaphyseal ROI extraction. IEEE Trans Med Imaging.

[13] Thodberg H, Kreiborg S, Juul A, Pedersen KD (2009). The BoneXpert method for automated determination of skeletal maturity. IEEE Trans Med Imaging.

[14] Spampinato C, Palazzo S, Giordano D, Aldinucci M, Leonardi R (2017). Deep learning for automated skeletal bone age assessment in X-ray images. Med Image Anal.

[15] Halabi SS, Prevedello LM, Kalpathy-Cramer J, Mamonov AB, Bilbily A, Cicero M, Pan I, Pereira LA, Sousa RT, Abdala N, Kitamura FC, Thodberg HH, Chen L, Shih G, Andriole K, Kohli MD, Erickson BJ, Flanders AE (2019). The RSNA pediatric bone age machine learning challenge. Radiology.

[16] Wibisono A, Saputri MS, Mursanto P, Rachmad J, Alberto, Yudasubrata ATW, Rizki F, Anderson E (2019) Deep Learning and Classic Machine Learning Approach for Automatic Bone Age Assessment. In: 2019 4th Asia-Pacific conference on intelligent robot systems, pages 235–240. IEEE, 2019.

[17] Iglovikov VI, Rakhlin A, Kalinin AA, Shvets AA (2018) Paediatric bone age assessment using deep convolutional neural networks. Deep Learning in Medical Image Analysis and Multimodal Learning for Clinical Decision Support, pages 300–308. Springer, 2018.

[18] Escobar M, González C, Torres F, Daza L, Triana G, Arbeláez P. (2019) Hand pose estimation for pediatric bone age assessment. In: International conference on medical image computing and computer-assisted intervention, pages 531–539. Springer, 2019

[19] Kim W, Kanezaki A, Tanaka M (2020). Unsupervised learning of image segmentation based on differentiable feature clustering. IEEE Trans Image Process.

[20] Howard A, Sandler M, Chen B, Wang W, Chen L, Tan M, Chu G, Vasudevan V, Zhu Y, Pang R, Adam H, Le Q (2019) Searching for MobileNetV3. In: IEEE/CVF international conference on computer vision (ICCV). IEEE , Seoul, Korea (South), Oct. 2019.

[21] Howard AG, Zhu M, Chen B, Kalenichenko D, Wang W, Weyand T, Andreetto M, Adam H. (2017). Mobilenets: efficient convolutional neural networks for mobile vision applications. https://arxiv.org/abs/1704.04861

[22] Sandler M, Howard A, Zhu M, Zhmoginov A, Chen LC. (2018) MobileNetV2: Inverted residuals and linear bottlenecks IEEE/CVF Conference on Computer Vision and Pattern Recognition (CVPR). IEEE, Salt Lake City, UT, Jun. 2018

[23] Hu J, Shen L, Albanie S, Sun G, Wu E (2019). Squeeze-and-excitation networks. IEEE Trans Pattern Analysis Mach Intelligence.

